# Prevalence of hepatitis B and C viruses infection among military personnel at Bahir Dar Armed Forces General Hospital, Ethiopia

**DOI:** 10.1186/s13104-015-1719-2

**Published:** 2015-12-01

**Authors:** Tigist Birku, Baye Gelaw, Feleke Moges, Abate Assefa

**Affiliations:** Department of Medical Microbiology, School of Biomedical and Laboratory Sciences, College of Medicine and Health Sciences, University of Gondar, Gondar, Ethiopia

**Keywords:** Hepatitis B virus, Hepatitis C virus, Military personnel

## Abstract

**Background:**

Military personnel are high-risk people for parenteral and sexually transmitted diseases such as hepatitis B virus (HBV) and hepatitis C virus (HCV). Data regarding HBV and HCV prevalence among military personnel in Ethiopia is limited. Hence, the study aimed to determine sero-prevalence and associated risk factors of HBV and HCV among military personnel at Bahir Dar Armed Forces General Hospital, Ethiopia.

**Methods:**

A cross-sectional study was conducted in a total of 403 military personnel from February to May 2015. Socio-demographic characteristics and risk factors were collected through face to face interview using structured questionnaire. HBV and HCV infection was determined using HBsAg and anti-HCV antibody rapid tests. Logistic regression analysis was employed to assess possible risk factors for HBV and HCV infections.

**Results:**

The sero-prevalence of HBV and HCV infection were 4.2 and 0.2 %, respectively. None of the study subjects were co-infected with HBV and HCV. Higher prevalence of HBV infection (11.3 %) was observed in the age group of 40 and above. Being at the age of 40 years and above (COR 7.6; 95 % CI 2.0–29.0, p = 0.003), history of nose piercing (COA 5.9; 95 % CI 1.2–29.9, p = 0.033) and sexually transmitted infection (COR 4.3; 95 % CI 1.1–16.4, p = 0.03) were significantly associated with these viral hepatitis infections.

**Conclusion:**

Intermediate prevalence of HBV and low prevalence of HCV were observed among military personnel. Strengthening HBV screening strategies among military personal may further reduce these viral diseases.

## Background

Viral hepatitis is an inflammation of the liver due to viral infections. The most common types of viruses that cause viral hepatitis are hepatitis B virus (HBV) and hepatitis C virus (HCV) [1]. HBV and HCV share some common mode of transmission such as parenteral route and unsafe sexual intercourse. Viral hepatitis is significant public health problem especially in resource-poor settings. Annually about 500,000–700,000 people die as a result of HBV infection and more than 350,000 people from HCV-related liver diseases [2–4]. Worldwide, more than 240 million people are chronically infected with HBV [5, 6] and 150 million people with HCV [2].

In Ethiopia, although nationwide survey report is lacking, an estimated prevalence of 10–15 % HBV infection and 2–5 % HCV infection were reported. More than 60 % of chronic liver disease and up to 80 % of hepato-cellular carcinoma were caused by HBV and HCV chronic infections [7, 8]. In addition, the prevalence of viral hepatitis showed great variability among different risk groups that should be taken into consideration when designing a more appropriate epidemiological investigation. In this regard, 4.11–6.2 % sero-prevalence of HBV has been reported among blood donors [9–11], 5.6 % among HIV infected individuals [12], 4.7 % among medical waste handlers [13], 10.9 % among street dwellers [14] and 10.4 % among prisoners [15]. The prevalence of HCV infection was reported 0.63–1.7 % among blood donors [9–11], 0.7 % among medical waste handlers [13] and 5 % among HIV positive individuals [12].

In most cases, military people live in military camps which may contribute to predispose them to HBV and HCV transmission through some common routes. The risk of sharing utensils such as hair-brushes, combs, razors and toothbrushes is common among people living in groups that can facilitate transmission of the viruses [16]. Moreover, usually soldieries travel from place to place for different professional reasons and stay longer apart from their family. This may force soldiers to have multiple sex partners that can expose them for different sexually transmitted infections (STIs) including HBV and HCV. Although several studies reported the prevalence of HBV and HCV infections among different risk groups, so far there is no published data about viral hepatitis prevalence among military people in the Amhara regional state of Ethiopia. Thus, this study primarily aimed to determine the sero-prevalence of HBV and HCV infections and associated risk factors among military personnel at Bahir Dar Armed Forces General Hospital, Ethiopia.

## Methods

### Study setting and design

The study was conducted at Bahir Dar Armed Forces General Hospital found in Bahir Dar town from the 1st of February to the 30th of May 2015. The town is located 578 km far from Addis Ababa. The hospital currently provides health service for more than 9000 military and military families. It has 5 wards with 200 beds for inpatient service. All military persons attending the hospital were eligible for the study. The sample size was determined using single population proportion formula with the assumption of 95 % confidence interval (CI), 5 % marginal error and 50 % prevalence of HBV and HCV infection because there was no previous similar study and 5 % none response rate. Therefore, the final sample size was determined 403. During the study period, a total of 1320 military patients were attended in the hospital and the study participants were recruited using systematic random sampling method.

### Data collection

Socio-demographic characteristics and associated risk factors for HBV and HCV infections were collected using structured questionnaire by trained health officer. The study variables included in this study were age, sex, marital status, work experience, history of hospitalization, blood transfusion, surgical procedure, ear/nose piercing, dental extraction, tattooing, STI, multiple sexual partner, family history of liver disease, sharing of shaving blade or nail cutter. Three milliliters of venous blood was collected from each study participant by trained laboratory technologist. Serum was separated by centrifugation at 3000 rpm for 5 min. Each serum was subjected to HBsAg and anti-HCV antibody rapid tests (Shanghai Eugene Biotech co., Ltd) following the manufacturer’s instruction. To ensure the quality of data positive and negative control sera were run following the manufacturer recommendation of the kit.

### Data analysis

Data was checked and cleared before entry. Data was entered and analyzed using SPSS version-20 computer software. Descriptive statistics were used to analyze socio-demographic characteristics, prevalence of HBV and HCV infections. Logistic regression analysis was employed to assess the possible associated factors of HBV and HCV infections. Odds ratio (OR), confidence interval (CI) and *p* value were computed to assess the presence and degree of association between dependent and independent variables. P-value < 0.05 was considered statistically significant for all cases.

### Ethical consideration

Ethical approval was obtained from School of Biomedical and Laboratory Sciences, College of Medicine and Health Sciences, University of Gondar ethical review committee. Official permission was obtained from Bahir Dar Armed Forces General Hospital higher management. Written informed consent was taken from each study participant and confidentiality was maintained at each level of the study. Individuals found HBV and/or HCV positive were linked to physician for appropriate management.

## Results

### Socio-demographic characteristics

A total of 403 military personnel were enrolled in this study. The majority of the study subjects 362 (89.8 %) were male and the mean age of the study participants was 32.6 ± 7 SD years. More than half of the military personnel (53.3 %) were married and only 10 (2.5 %) were widowed or divorced. Data on educational status showed that 194 (48.1 %) military personnel completed college or higher degree and only 26 (6.5 %) had elementary school education. The majority of the soldiers (79.2 %) were recruited from urban settings and 325 (80.6 %) of them were orthodox Christian by religion (Table 1).

**Table 1:** Socio-demographic characteristics of military personnel and sero-prevalence of HBV and HCV at Bahir Dar armed force general hospital, 2015

Variable	Total n (%)	HBV		HCV	
		Positive n (%)	Negative n (%)	Positive n (%)	Negative n (%)
Sex					
Female	41 (10.2)	2 (4.9)	39 (95.1)	0	41 (100)
Male	362 (89.8)	15 (4.2)	347 (95.8)	1 (0.3)	361 (99.7)
Age (years)					
20–29	160 (39.7)	3 (1.9)	157 (98.1)	0	160 (100)
30–39	172 (42.6)	6 (3.5)	166 (96.5)	0	172 (100)
40+	71 (17.6)	8 (11.3)	63 (88.7)	1 (1.4)	70 (98.5)
Residence					
Urban	319 (79.2)	12 (3.8)	307 (96.2)	1 (0.3)	318 (99.7)
Rural	84 (20.8)	5 (6.0)	79 (94.0)	0	84 (100)
Educational status					
Elementary	26 (6.5)	0	26 (100)	0	26 (100)
High school	183 (45.4)	11 (6)	172 (93.9)	1 (0.5)	182 (99.5)
Collage+	194 (48.1)	6 (3.1)	188 (96.9)	0	194 (100)
Religion					
Orthodox	325 (80.6)	14 (4.3)	311(95.7)	1 (0.3)	324 (99.7)
Muslim	42 (10.4)	2 (4.8)	40 (95.2)	0	42 (100)
Others	36 (8.9)	1 (2.8)	35 (97.2)	0	36 (100)
Marital status					
Single	178 (44.2)	5 (2.8)	173 (97.2)	0	178 (100)
Married	215 (53.3)	12 (5.6)	203 (94.4)	1 (0.5)	214 (99.5)
Widowed	2 (0.5)	0	2 (100)	0	2 (100)
Divorced	8 (2.0)	0	8 (100)	0	8 (100)
Work experience (year)					
<10	179 (44.4)	5 (2.8)	174 (97.2)	0	179 (100)
10–20	183 (45.4)	10 (5.5)	173 (94.5)	1 (0.5)	182 (99.5)
20+	41(10.2)	2 (4.9)	39 (95.1)	0	41 (100)

### Sero-prevalence of HBV and HCV infection

The overall sero-prevalence of HBV and HCV infections was 18/403 (4.5 %) of which 17 (4.2 %) was positive for HBV and only one individual was positive for HCV. The point prevalence of HBV infection was 318.2/100,000 but that of HCV was 15.2/100,000. However, none of the soldiers were co-infected by HBV and HCV. Higher prevalence of HBV infection 8/71 (11.3 %) was found among soldiers with age ≥40 years. Moreover, the sero-prevalence of HBV infection was relatively higher among rural dwellers 5/84 (6 %) and married soldiers 12/215 (5.6 %) (Table 1).

### Risk factor analysis for hepatitis virus infection

The majority of military people had history of hospitalization (33.5 %) and dental extraction (28.8 %). Thirty-nine (9.7 %) soldiers had history of different surgical procedures and 14 (3.4 %) had multiple (more than two) sexual partners. Only 17 (4.2 %) soldiers had family history of liver disease. Bivariate logistic regression analysis showed that soldiers with an age ≥40 years (COR 7.6; 95 % CI 2.0–29.0, p = 0.003), history of nose piercing (COA 5.9; 95 % CI 1.2–29.9, p = 0.033) and history of STI (COR 4.3; 95 % CI 1.14–16.4, p = 0.03) were significantly associated with hepatitis virus infections. However, study variables such as history of hospitalization, tattooing, dental extraction, family history of liver disease, ear piercing, war related injury, history of blood transfusion, number of sexual partners and sharing of shaving blade or nail cutter were not associated with infection caused by these hepatitis viruses (Table 2).

**Table 2:** Bivariate regression analysis of risk factors association with hepatitis virus infection among military patients at Bahir Dar armed force general hospital, 2015

Variables	Viral hepatitis		COR (95 % CI)	P value
	Positive n	Negative n		
Sex				
Female	2	39	1	
Male	16	346	0.902 (0.20–4.10)	0.89
Age				
20–29	3	157	1	
30–39	6	166	1.89 (0.47–7.69)	
≥40	9	62	7.59 (1.99–28.99)	0.003
Current residence				
Urban	13	306	1	
Rural	5	79	1.49 (0.52–4.30)	0.461
Educational status				
Up to 12	12	197	1	
College and above	6	188	0.52 (0.19–1.42)	0.205
Religion				
Orthodox	15	310	1	
Muslim + others	3	75	0.83 (0.23–2.93)	0.768
Marital status				
Single	5	183	1	
Married	13	202	2.35 (0.82–6.74)	0.11
Work experience				
<10	5	173	1	
10–20	11	173	2.20 (0.75–6.46)	0.152
>20	2	39	1.77 (0.33–9.49)	0.503
Hospitalization				
No	11	257	1	
Yes	7	128	1.28 (0.48–3.37)	0.621
Tattooing				
No	13	321	1	
Yes	5	64	1.93 (0.67–5.60)	0.227
Dental extraction				
No	11	276	1	
Yes	7	109	1.61(0.61–4.26)	0.337
Surgical history				
No	14	350	1	
Yes	4	35	2.86 (0.89–9.15)	0.077
Number of sexual partner				
0	2	89	1	
1	14	284	2.19 (0.49–9.84)	0.305
**≥**2	2	12	7.41 (0 .95–57.64)	0.055
Family history of liver disease				
No	16	369	1	
Yes	2	16	2.88 (0.61–13.62)	0.181
Ear piercing				
No	15	326	1	
Yes	3	59	0.91 (.25–3.22)	0.877
Nose piercing				
No	16	376	1	
Yes	2	8	5.85 (1.15–29.9)	0.033
STI				
No	15	368	1	
Yes	3	17	4.33 (1.14–16.39)	0.031
War related injury				
No	15	326	1	
Yes	3	59	0.91 (.25–3.22)	0.877

0 no sexual partner, 1 one sexual partner, ≥2 sexual partner two and above

*COR* crude odds ratio, *CI* confidence interval, *STI* sexually transmitted infection

## Discussion

The result of the present study showed that the sero-prevalence of HBV infection among the military personnel was 4.2 %. The sero-prevalence of HBV infection was reported 3.0 % among delivering women in Ethiopian [17]. Another report showed a 4.11 and 4.7 % prevalence of HBV infection among blood donors and medical waste handlers [13]. On the other hand, higher prevalence of HBV infection was reported from Gondar town street dwellers (10.9 %) [14] and Woldia prisoners (10.4 %) [15]. Higher prevalence of HBV infections was also observed in some other African countries among different population groups. For instance, a prevalence of 8.2 % was reported in Sudan among healthy people [18], 23 % in Nigeria among prisoners [19] and 11.9 % among HIV positive patients in Nigeria [20]. The discrepancies in the prevalence of HBV infection among different study groups might be explained by the variation in predisposing conditions. Military people and street dwellers have higher opportunity to be exposed to infections transmitted through sexual intercourse and parenteral routes. In-contrast, the prevalence of HBV infection found in the current study was relatively higher than the findings reported from Pakistan 2.9 % [21] and Greece 0.32 % among military groups [22].

The prevalence of HBV and HCV infection found in the current study can be graded intermediate and low according to WHO criteria [23]. The prevalence of HBV infection can be graded high when the prevalence is >8 %, intermediate when the prevalence is between 2 and 8 % and low when the prevalence is <2 % [24]. Hepatitis C virus infection can be also graded high, moderate or low when the prevalence is >3.5, 1.5–3.5 % and <1.5 % respectively [25]. In the current study, only one study subject (0.2 %) was positive for HCV infection. This prevalence is nearly similar to previous reports among medical waste handlers (0.7 %) in Gondar, Ethiopia [13], among apparently health people in Nigeria (0.8 %), and Morocco (0.62 %), and among blood donors in Jordan (0.8 %) and in Pakistan (0.89 %) [26–29]. Lower prevalence of HCV infection was also reported (0.82 %) among military groups in Kabul military training center [30]. On the other hand, relatively higher prevalence of HCV infection (1.7 %) was reported in Pakistan among military groups [21].

The current study showed significantly higher (11.3 %) prevalence of HBV infection among solders within the age group of ≥40 years. Similarly, in China significantly different HBV clinical and virological characteristics among patients with chronic HBV infection at different age groups was reported. Among patients at the age of ≥40 years, there were markedly more reactivation cases than in any other groups [31]. Moreover, it was also observed that by the age of 40 years, 87 % of the Nigerian population has at least one HBV serologic markers [32].

In the current study, history of hospitalization, tattooing, dental extraction, ear piercing, war related injury, and sharing of shaving blade or nail cutter were not significantly associated with hepatitis virus infections. Contrary to this finding, a significant association between histories of blood transfusion, body tattooing, surgery and unsafe injection for HBV infections was reported among pregnant women at Bahir Dar, Ethiopia [33]. A similar study, which was conducted in Egypt, identified these variables as significant risk factors for HBV infection. Study participants with body tattoo on any part of their body were 5.7 times more likely to be HBsAg positive [34]. Furthermore, reports from Bamako, Mali indicated that body tattooing was a significant risk factor for HBV infection [35]. However, in this study history of STI showed significant association with HBV infection. Evidences demonstrate that military people are considered to be a gateway group for STIs [36]. Absence of HBV and HCV co-infection in this study is supported by a study conducted among blood donors in the Amhara and Tigray national state of Ethiopian [11]. A report from Nigeria among prisoners was found also only 0.07 % HBV and HCV co-infected individuals [19]. Therefore, HCV infection is uncommon among HBV positive patients and some authors describe mutual inhibition [37].

## Conclusion

The prevalence of HBV and HCV infection among soldiers at Bahir Dar Armed Forces General Hospital was intermediate and low respectively. Higher prevalence of HBV infection was observed among soldiers with an age of ≥40 years. Moreover, older age, history of STI and nose piercing were significantly associated with HBV and HCV infections. Strengthening HBV screening strategies among military personal may further reduce these viral diseases.
